# CLINICAL DEMOGRAPHIC CHARACTERISTICS OF TOTAL KNEE ARTHROPLASTY IN A UNIVERSITY HOSPITAL

**DOI:** 10.1590/1413-785220162406159988

**Published:** 2016

**Authors:** José Miguel Francisco da Silva Souza, Ricardo dos Santos Ferreira, Alexandre José Pereira de Lima, Airton César Pereira de Sá, Paulo Cezar Vidal Carneiro de Albuquerque

**Affiliations:** 1. Universidade Federal de Pernambuco, Hospital das Clínicas, Orthopedics and Traumatology Service, Recife, PE, Brazil.

**Keywords:** Knee, Arthroplasty, Epidemiology, Infection.

## Abstract

**Objective:**

To assess socio-demographic characteristics of patients undergoing total knee arthroplasty (TKA) in a public university hospital, evaluating the outcome infection and associated factors.

**Method:**

A retrospective study was carried out with 78 patients undergoing TKA, from 2013 to 2014. The socio-demographic and clinical characteristics of the patients were collected. Comparison between infected and non-infected patients was performed to find out which variables were possibly associated to this complication.

**Result:**

Of 81 arthroplasties performed, patients were older (mean age 64 years), women (79%), with primary osteoarthritis as main etiology (87.6%) and most had comorbidities (82.7%). Infection occurred in 16% of patients, and this outcome associated with age older than 65 years (p=0.023) and the occurrence of deep vein thrombosis (p=0.027).

**Conclusion:**

Patients undergoing TKA are mostly elderly women with primary osteoarthritis in the knee and comorbidities who developed infection in 16% of cases. More studies need to be conducted aimed at creating specific protocols in order to improve the quality of clinical practice. Level of Evidence III, Retrospective Comparative Study.

## INTRODUÇÃO

Since the nineteenth century, the treatment of serious knee joint diseases with joint replacement (arthroplasty) has been recognized and has received deserved attention. In 1860, Verneviul^1^ suggested interposing of soft tissue for reconstructing the knee joint. In the twentieth century, total knee arthroplasty (TKA) has greatly evolved, due to the development of inorganic materials suitable for joint interposition and improvement of the surgical technique, driven mainly by the studies of Campbell^2^ and McKeever.^3^ TKA is used to treat refractory chronic pain mostly due to primary arthrosis.^4^ ^,^ ^5^ TKA is a major surgery and subject to post-operative complications and infection is one of the worst and most feared complication, representing an actual challenge to the orthopedic surgeon, since it is difficult and lengthy to treat.^6^ The infections after knee arthroplasty represent an estimated economic impact of US$ 50,000 per patient in the US.^7^

To succeed the treatment of infection post total knee arthroplasty, early and accurate diagnosis should be immediate. Therefore, it is essential that all patients complaining of pain at the site of a total knee arthroplasty are evaluated for the possible presence of infection.^8^ The surgical site infection can be classified as superficial or deep; those involving only skin and subcutaneous tissue are considered superficial and those involving deep tissue incision, such as fascia and muscle are considered deep infections.^9^

In the acute form of infection, constant local pain, heat, swelling, redness and joint effusion are evident and almost always caused by Staphylococcus aureus and gram negative bacilli (Escherichia coli, Proteus sp, Pseudomonas aeruginosa).^10^ Some laboratory tests should be requested, such as erythrocyte sedimentation rate and the level of C-reactive protein (CRP) when infection is a suspicion.^11^ Carvalho Junior et al.^12^ demonstrated the correlation of CRP and erythrocyte sedimentation rate levels, showing that these go back to normal levels 30-80 days after surgery. The correlation of physical examination, laboratory tests and imaging tests are essential for the diagnosis of prosthesis infection.^11^ ^,^ ^12^

The prevalence of primary TKA infection is between 0.4% and 2% in the US.^13^ ^,^ ^14^ Malinzak et al.^15^ reported a 0.51% infection rate in 8,494 hip and knee arthroplasties, moreover, they found as risk factors for infection: obesity, early age and diabetes *mellitus*. In Spain, the prevalence of TKA infection is 3-4%.^16^ In Brazil, some authors have shown that the prevalence of superficial infection of TKA is 1.2%.^4^

The study is justified by the need to establish a diagnostic protocol and early treatment to reduce complications to the patient and costs to public health systems.

The aim of this study was to establish the socio-demographic profile of patients undergoing TKA performed in a public hospital, evaluating the outcome infection and associated factors.

## MATERIALS AND METHODS

This study was approved by the Research Ethics Committee of Hospital Público Universitário under the protocol number 1007986/CAAE 42681815.4.0000.5208. All authors signed a Free and Informed Consent Form.

A retrospective cross-sectional study included 78 adult patients undergoing knee arthroplasty operated by orthopedic surgeons of a public university hospital from January 2013 to December 2014.

The diagnosis of TKA infection occurred during hospitalization and outpatient consultation during the follow-up period between six and 30 months.

Exclusion criteria were incomplete medical records, patients unidentified in the hospital database and infections acquired in other hospitals. Data from medical record was collected and stored in a Microsoft Office Excel 2007 spreadsheet. The variables age, gender, etiology, comorbidities, use of prophylactic antibiotics, complications, primary surgery and revision were collected for each patient. The qualitative variables were described as frequencies and percentages. To evaluate the association between two dichotomous qualitative variables the Fisher's exact test was employed with the statistical software Epi Info. P-Values <0.05 were considered statistically significant.

## RESULTS

**Figure 1:**
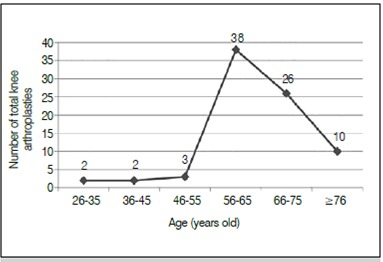
Number of total knee arthroplasties performed by age groups in 2013-2014.

Eighty one total arthroplasties were performed, 78 unilateral primary TKA, three bilateral primary TKA in two stages and a review surgery. As to gender, 17 patients (20.9%) were male and 64 (79.1%), female. Regarding etiology, only osteoarthritis affected 71 (87.65%) patients. The age range was between 29-84 years old (mean 64 years). (Figure 1)

As complications, we diagnosed 13 (16.04%) infections, eight (9.88%) involving deep tissue and five (6.17%) involving superficial tissues. Among the 81 arthroplasties, five (6.17%) were preceded by deep vein thrombosis (DVT), all cases confirmed by Doppler ultrasound, one case (1.23%) had compartment syndrome and one patient (1.23%) died.

Considering the 13 patients who developed TKA infection, nine (69.23%) were female, 10 (76.92%) were over 65 years of age (p = 0.023). Regarding the comorbidities of infected individuals, 12 (92.31%) presented some associated clinical disease. Among these diseases, 10 (76.92%) patients had hypertension and two (15.38%) had DM. Even among those who presented infections, one patient had rheumatoid arthritis (RA) and had gout (15.38%). However, among all surgeries, five (6.17%) had rheumatoid arthritis. Prophylactic antibiotic was administered 30 min before the surgical incision in 12 (92.31%) patients. The recommendation of the Hospital Infection Control Committee was to start antibiotic prophylaxis 30 min before the surgical incision and maintain it for 24h postoperatively. However, some surgeons have chosen to increase it to 48h postoperatively. There was no difference between those treated for 24h and 48h. The antibiotic used in the prophylaxis was 2g cefazolin before the incision and 1g each 8h postoperatively. If cefazolin was not available, 2g cephalothin was administered before incision and 1g every 6h postoperatively. Deep vein thrombosis, considered the second most common complication, preceded three (23.08%) of TKA infections (p=0.027). (Table 1)

**Table 1:** Analysis results showing the association of the main variable infected total knee arthroplasty with other variables analyzed, and *p*-values.

Variables	Infected total knee arthroplasty n (%)	*p* value
Gender		
Female	9 (69.23)	0.23
Male	4 (30.77)	-
Age (years old)		
> 65	10 (76.92)	0.023
≤ 65	3 (23.08)	-
Comorbidities		
Systemic hypertension	10 (76.92)	0.5
Diabetes *mellitus*	2 (15.38)	0.6
Rheumatic disease	2 (15.38)	0.31
Prophylactic antibiotic therapy		
Yes	12 (92.31)	0.62
No	1 (7.69)	-
Infection		
Deep	8 (61.54)	-
Superficial	5 (38.46)	-
Deep vein thrombosis	3 (23.08)	0.027
Compartment Syndrome	1 (7.69)	-
Death	1 (7.69)	-

## DISCUSSION

The mean age of patients undergoing TKA reported by other researchers varied between 65 and 71 years old,^4^ ^,^ ^17^ somehow above the mean age in the present study of 64 years old. The preferential involvement of the elderly is related to cumulative exposure to various risk factors and biological changes that occur with aging, such as thinning of the cartilage, decreased muscle strength and oxidative stress.^18^ This study showed that women preferably developed osteoarthritis, which is consistent with the international literature.^18^ This fact is probably related to menopause, which interferes with the female hormone levels. Regarding etiology, Piano et al.^5^ performed a Brazilian study that showed that the diagnostic profile of patients reached 92.4% only for osteoarthritis, as another study^4^ revealed a smaller percentage of 84.9% of primary knee osteoarthritis, which is similar to another study with 87.65%.

The level of TKA infection of this study (16.04%) was higher than others found in the literature.^13^ ^-^ ^16^ ^,^ ^19^ ^,^ Moreover, the level of superficial infections was up to five times higher, and deep infections exceeded the level found in the national^4^ ^,^ ^19^ and international literature.^17^

Considering this worrisome scenario, it was decided to temporarily suspend TKA procedures and a protocol was elaborated by surgeons and the Hospital Infection Control Committee, which addressed various requirements that were not a routine procedure previously before considering TKA surgeries. Among these requirements are urine culture tests; if the result showed abnormal, the patient was treated with antibiotics and the test repeated. The surgical environment must be under laminar air flow; all surgical clothing should be waterproof and disposable; patients should be medicated with mupirocin nasal solution three days before surgery, in order to obtain nasal decolonization. Furthermore, antibiotic therapy must start 40 min prior to surgical incision with 2g cefazolin for patients weighting up to 120 Kg and 3g for heavier patients. The dose is repeated every 2h during the surgery and maintained every 8h for 24h postoperatively.

Brazilian researchers^11^ showed that females were preferentially affected among patients with TKA infection, with a prevalence of 65.51%, a result similar to the present study (69.23%). Furthermore, we found a significant associations of TKA infection with the age over 65 years (p = 0.023), unlike the results of Pinto et al.,^19^ which found no statistically significant association. Five patients (6.17%) submitted to TKA developed deep vein thrombosis and three of them had infection (p = 0.027), a much higher rate than that observed by Lenza et al.^4^ and Xu et al.^17^ Only one patient of this study had died, almost half the prevalence found by Pinto et al.;^19^ however, higher than Lenza et al.,^4^ who had no deaths among patients undergoing TKA.

Prophylactic antibiotics did not statistically correlate to infection prevention (p = 0.62), however, literature data is consistent regarding the indication of chemoprophylaxis to prevent TKA infection.^4^ ^,^ ^5^ ^,^ ^12^

Systemic hypertension was the most prevalent comorbidity among infected patients, a result similar to other studies.^4^ ^,^ ^5^ Patients with diabetes *mellitus* had no statistically significant association with TKA infection (p = 0.60). It is important to note that Malinzak et al.^15^ concluded that diabetic patients are 3.1 times more likely to have TKA infection. Just as diabetes *mellitus*, rheumatic diseases had a similar prevalence (15.38%) among patients with TKA infection, but there was no statistically significant association. Only one patient had rheumatoid arthritis among those infected, however, considering all 81 arthroplasties, 6.17% had rheumatoid arthritis, five times more prevalent than in the study by Lenza et al.,^4^ and almost three times more prevalent than in the study by Pinto et al.^19^

## CONCLUSION

Patients undergoing TKA are mostly elderly women, with primary knee osteoarthritis and comorbidities that evolve to infection in 16% of cases. TKA infection had as statistically significant risk factors age over 65 years and deep vein thrombosis. These results should serve to improve prevention of deep vein thrombosis. More studies are needed aiming to create specific protocols in order to improve the quality of clinical practice with consequent reduction of postoperative complications.

## References

[1] Verneviul A (1860). De lá création dune fausse articulation par section ou ressection partielle de los maxillaire inférieur, comme moyen de rémedier a lankylose vraie ou fausse de la machoire inférieure. Arch Gen Med.

[2] Campbell WC (1940). Interposition of vitallium plates in arthroplasties of knee: preliminary report. Am J Surg.

[3] Mckeever DC (1960). Tibial plateau prosthesis. Clin Orthop Relat Res.

[4] Lenza M, Ferraz SB, Viola DCM, Garcia RJ, Cendoroglo M, Ferretti M (2013). Epidemiologia da artroplastia total de quadril e de joelho: estudo transversal. Einstein.

[5] Piano LPA, Golmia RPA, Scheinberg M (2010). Artroplastia total de quadril e joelho: aspectos clínicos na fase perioperatória. Einstein.

[6] Mulvey TJ, Thornhill TS, Insall JN (2001). Infected total knee arthroplasty.

[7] Sculco TP (1995). The economic impact of infected joint arthroplasty. Orthopedics.

[8] Ayers DC, Dennis DA, Johanson NA, Pellegrini VD (1977). Common complications of total knee arthroplasty. J Bone Joint Surg Am.

[9] Ministério da Saúde (BR). Agência Nacional de Vigilância Sanitária. Gerência de Vigilância e Monitoramento em Serviços de Saúde (2013). Critérios Diagnósticos de Infecção Relacionada à Assistência à Saúde.

[10] Cho WS, Jeong YG, Park JH, Shin HK, Kim KY, Seon MW (2001). Treatment of infected TKRA. J Korean Orthop Assoc.

[11] D'Elia CO, Santos ALG, Leonhardt MC, Lima ALLM, Pécora JR, Camanho GL (2007). Tratamento das infecções pós artroplastia total de joelho: resultados com 2 anos de seguimento. Acta Ortop Bras.

[12] Carvalho LH, Temponi EF, Badet R (2013). Infecção em artroplastia total de joelho: diagnóstico e tratamento. Rev Bras Ortop.

[13] Mahomed NN, Barrett J, Katz JN, Baron JA, Wright J, Losina E (2005). Epidemiology of total knee replacement in the United States Medicare population. J Bone Joint Surg Am.

[14] Peersman G, Laskin R, Davis J, Peterson M (2001). Infection in total knee replacement: a retrospective review of 6489 total knee replacements. Clin Orthop Relat Res.

[15] Malinzak RA, Ritter MA, Berend ME, Meding JB, Olberding EM, Davis KE (2009). Morbidly obese, diabetic, younger, and unilateral joint arthroplasty patients have elevated total joint arthroplasty infection rates. J Arthroplasty.

[16] Ariza J, Gorane G, Murillo O (2008). Infecciones relacionadas con las prótesis articulares. Enferm Infecc Microbiol Clin.

[17] Xu GG, Sathappan SS, Jaipaul J, Chan SP, Lai CH (2008). A review of clinical pathway data of 1,663 total knee arthroplasties in a tertiary institution in Singapore. Ann Acad Med Singapore.

[18] Zhang Y, Jordan JM (2010). Epidemiology of osteoarthritis. Clin Geriatr Med.

[19] Pinto CZS, Alpendre FT, Stier CJN, Maziero ECS, Alencar PGC, Cruz EDA (2015). Caracterização de artroplastias de quadril e joelho e fatores associados à infecção. Rev Bras Ortop.

